# The CD36-PPARγ Pathway in Metabolic Disorders

**DOI:** 10.3390/ijms19051529

**Published:** 2018-05-21

**Authors:** Loïze Maréchal, Maximilien Laviolette, Amélie Rodrigue-Way, Baly Sow, Michèle Brochu, Véronique Caron, André Tremblay

**Affiliations:** 1 Research Center, CHU Sainte-Justine, Montréal, QC H3T 1C5, Canada; loize.marechal@umontreal.ca (L.M.); maximilien.laviolette-brassard@umontreal.ca (M.L.); amelierway@aol.com (A.R.-W.); baly.sow@umontreal.ca (B.S.); veronique.caron.4@gmail.com (V.C.); 2 Department of Physiology, Faculty of Medicine, University of Montreal, Montréal, QC H3T 1J4, Canada; michele.brochu@umontreal.ca; 3 Department of Biochemistry and Molecular Medicine, Faculty of Medicine, University of Montreal, Montréal, QC H3T 1J4, Canada; 4 Centre de Recherche en Reproduction et Fertilité, University of Montreal, Saint Hyacinthe, QC J2S 7C6, Canada; 5 Department of Obstetrics & Gynecology, Faculty of Medicine, University of Montreal, Montréal, QC H3T 1C5, Canada

**Keywords:** scavenger receptor, PPAR nuclear receptors, PGC-1, fatty acid oxidation, energy metabolism, GHRP, hexarelin, atherosclerosis, insulin resistance

## Abstract

Uncovering the biological role of nuclear receptor peroxisome proliferator-activated receptors (PPARs) has greatly advanced our knowledge of the transcriptional control of glucose and energy metabolism. As such, pharmacological activation of PPARγ has emerged as an efficient approach for treating metabolic disorders with the current use of thiazolidinediones to improve insulin resistance in diabetic patients. The recent identification of growth hormone releasing peptides (GHRP) as potent inducers of PPARγ through activation of the scavenger receptor CD36 has defined a novel alternative to regulate essential aspects of lipid and energy metabolism. Recent advances on the emerging role of CD36 and GHRP hexarelin in regulating PPARγ downstream actions with benefits on atherosclerosis, hepatic cholesterol biosynthesis and fat mitochondrial biogenesis are summarized here. The response of PPARγ coactivator PGC-1 is also discussed in these effects. The identification of the GHRP-CD36-PPARγ pathway in controlling various tissue metabolic functions provides an interesting option for metabolic disorders.

## 1. Introduction

In years to come, metabolic defects are predicted to remain one of the principal causes of death and disability in industrialized countries, and their occurrence is seen to be increasing in several developing countries. Excess body weight is considered a major risk factor for metabolic disorders, and the epidemic of pre-obese and obese conditions and type 2 diabetes and their increasing prevalence in children indicate that these pathologies will continue to impact human health [1,2]. Hence, the mechanisms underlying excessive fat storage and its clinical complications remain a challenge to understand and treat.

The liver, skeletal muscle and fat tissue are known as the major sites for the central control of adaptive metabolic regulation of fatty acids (FA) in the body, playing a critical role in maintaining normal glucose and lipid homeostasis. In the condition of surpassed lipid storage, the normal fatty acid metabolism is disrupted and consequent build-up of fat accumulation occurs in non-adipose depots such as the liver, pancreatic islets, muscle, and myocardium. Such accumulation contributes to eliciting a number of metabolic defects, such as dyslipidemia, atherosclerosis, hypertension, and type 2 diabetes [3,4,5]. While numerous therapeutic strategies are being developed and used in clinics in our attempt to correct the various conditions associated to metabolic dysfunctions, targeting the peroxisome proliferator-activated receptors (PPARs) undoubtedly remains an important option of treatment.

## 2. The Peroxisome Proliferator-Activated Receptors (PPARs): Fatty Acid Sensors Controlling Metabolism

The PPARs consist of three isotypes, PPARα (NR1C1), PPARβ/δ (NR1C2), and PPARγ (NR1C3), which belong to the nuclear receptor family of ligand-activated transcription factors [6]. PPARs variously bind mono- and polyunsaturated fatty acids and derivatives such as eicosanoids to control the transcription of many genes that govern lipid metabolism [7]. Once activated, they heterodimerize with the nuclear receptor RXR (NR2B family) to bind DNA and modulate target gene transcription [8]. PPARα is a target of the hypolipidemic fibrate drugs and a major activator of FA oxidation in the liver, heart and brown adipose tissue [9,10]. PPARβ/δ is ubiquitously expressed and shares similar functions with PPARα in promoting FA oxidation in metabolic tissues such as skeletal muscle, liver and heart [10,11]. PPARγ is most highly expressed in metabolic tissues including white and brown adipose tissue, where it is a master regulator of whole-body lipid metabolism, adipogenesis, and insulin sensitivity [9,12]. Compared to the other PPARs, PPARγ responds poorly to native fatty acids, while oxidized fatty acid derivatives contained in circulating oxidized low-density lipoproteins (oxLDL) elicit a strong PPARγ activation [13]. PPARγ activity is regulated by transcriptional coactivators such as PGC-1, and also by post-translational modifications often independent of ligand binding, such as phosphorylation, ubiquitination, and SUMOylation [14,15]. In addition to its role in lipid and glucose metabolism, PPARγ has been involved in macrophage cholesterol metabolism and inflammatory response and also plays a major role in mitochondrial physiology and energy metabolism [16,17,18].

Because of its potent insulin-sensitizing activity, PPARγ has been recognized as a major therapeutic target with the identification of thiazolidinediones (TZDs) as high-affinity ligands [19,20]. TZDs are currently used to correct circulating glucose levels in type 2 diabetes patients [21,22]. However, the clinical efficacy of TZDs on insulin sensitivity has become limited [9,23]. This is partly due to their side effect of stimulating adiposity by upregulating PPARγ target genes, such as fatty acid synthase (FAS) and scavenger receptor CD36 involved in FA formation and storage [24,25]. More importantly, serious health issues have restricted the use of TZDs lately. As a result, some TZDs have been withdrawn from clinics due to life-threatening hepatic toxicity, while serious safety warnings were recently issued for others [26,27,28]. While strategies to develop safer PPAR pan/dual agonists are of continuous interest [29,30], it has become a fundamental priority to identify other treatment strategies in order to avoid the adverse effects of PPAR ligands while keeping the benefits of correcting whole body glucose and cardiovascular dysfunctions. Our recent identification of PPARγ as a new target of CD36 signaling might feed into the development of potential alternatives in the beneficial control of lipid metabolism.

## 3. The Growth Hormone Releasing Peptide (GHRP) Family

Growth hormone releasing peptides (GHRP; also known as growth hormone secretagogues) are a family of synthetic peptides and peptidomimetic agonists initially designed to promote growth hormone secretion in GH-deficient patients. However, despite tremendous effort at designing efficacious GHRPs that will exhibit elevated oral bioavailability and induce the pulsatile release of GH, low-cost recombinant GH remains the treatment of choice for GH-deficient patients. Yet, the use of GHRPs in human subjects appears relatively safe, highlighting positive effects on children growth velocity, increased lean mass, decreased bone turnover, and improved cardiac function [31,32,33]. However, studies are still needed to address the long-term impact of GHRPs and their benefits in diverse clinical scenarios.

GHRP-6 was the first GH-releasing efficient hexapeptide designed, which was then modified as GHRP-2, but their poor oral bioavailability and short-lasting effect have limited their use [34,35]. To address this drawback, additional compounds were designed, including MK-0677, a non-peptidic sulfonamide derivative [36], and hexarelin, also referred to as examorelin or EP-23905 [37,38]. Hexarelin (His-d-2MeTrp-Ala-Trp-d-Phe-Lys-NH_2_) differs from GHRP-6 by having d-Trp substituted by d-2-methyl-Trp, making hexarelin biologically more stable with greater GH release activity than GHRP-6 and the first orally active GHRP. Studies in humans have shown that hexarelin was efficient and well tolerated, eliciting a substantial and dose-dependent elevation in plasma GH concentrations, while causing minor sleep problems as side effects [33,39]. Because of the highly vascularized nasal cavity, intranasal administration was also implemented for hexarelin that further improved its bioavailability and efficacy as a therapeutic tool for GH deficiency [40]. Mainly due to their potent GH-releasing activities, hexarelin and other GHRPs, such as GHRP-2 and GHRP-6, are used to enhance athletic performance. This resulted in the implementation of routine GHRP screening since the 2014 Olympics in Sochi and the banning of its use by the World Anti-Doping Agency [41,42].

## 4. Central vs. Peripheral Actions of GHRPs

The receptor that mediates the response to GHRPs was initially identified as the growth hormone secretagogue receptor GHS-R1a, a member of the G protein-coupled receptor family [43]. GHS-R1a exhibits high-affinity binding toward GHRPs and is highly expressed in the anterior pituitary gland and hypothalamus, consistent with its role in regulating central GH release. A second isoform, GHS-R1b, was also identified but represents a truncated GHS-R receptor devoid of signal transduction activity and thought to act as a dominant-negative form of GHS-R1a through heterodimer formation [44]. Interestingly, ghrelin was later discovered as the endogenous ligand of GHS-R1a, which was then renamed the ghrelin receptor [45]. Ghrelin is an acetylated 28 amino acid hormone initially isolated from the stomach, which promotes central release of GH in somatotroph cells and induces orexigenesis [46,47,48]. Also consistent with a role in fat and energy metabolism [31,49], decreased circulating ghrelin levels were reported in obese children, increasing their prevalence to insulin resistance and metabolic syndrome [50,51,52].

Peripheral distribution of GHS-R1a has been reported, supporting physiological effects of GHRPs independently from GH release. Tissues such as vascular endothelium, heart, adrenals, monocytes/macrophages, β pancreatic cells, and bone were shown to express GHS-R1a [53,54,55]. Consistent with such a GH-independent role, peripheral ghrelin actions have been linked to clinical implications of cardiovascular disease, insulin resistance, and obesity [31,56,57,58,59]. Likewise, GH-independent effects on fat metabolism, cardioprotection, hemodynamic control, and bone cell differentiation have been reported for GHRPs [60,61,62,63,64,65,66]. Hence, such peripheral effects of GHRPs are thought to play important roles in energy homeostasis, adiposity and vascular integrity and identify the GHRPs as highly promising therapeutic targets in metabolic diseases.

## 5. Scavenger Receptor CD36, a Target of Hexarelin

**Figure 1:**
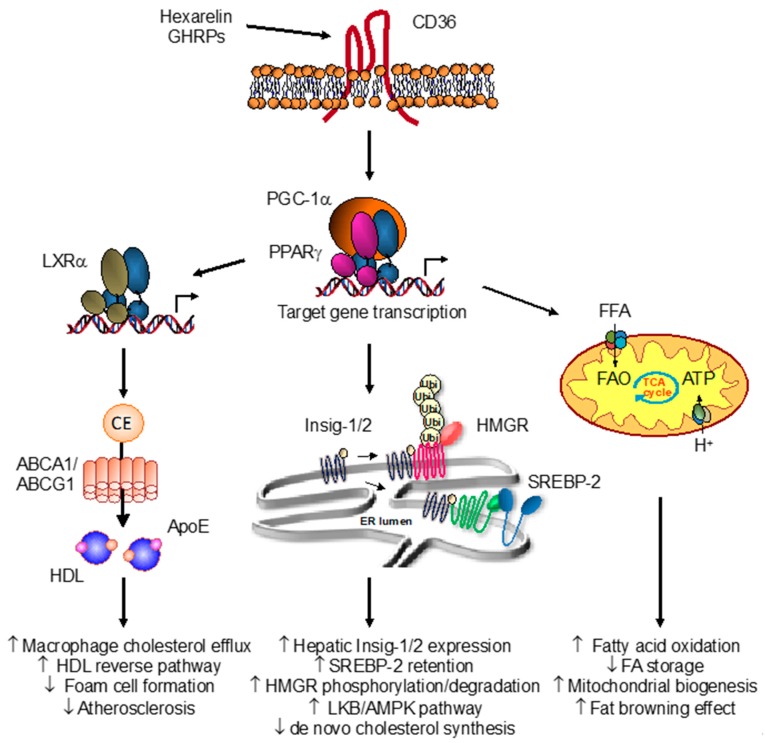
Overview of the growth hormone releasing peptide (GHRP)-peroxisome proliferator-activated receptor-gamma (PPARγ) pathway in lipid and energy metabolism. The interaction of hexarelin with the scavenger receptor CD36 promotes the transcriptional activation of nuclear receptor PPARγ and target gene profiling involved in metabolism. In macrophages, hexarelin and other GHRPs induce a molecular cascade involving nuclear liver X receptor LXRα and expression of apolipoprotein E (apoE) and sterol transporters ABCA1 and ABCG1. Such activation of the PPARγ-LXRα-ABC metabolic pathway increases cholesterol efflux, resulting in enhanced HDL reverse cholesterol transport and regression of atherosclerosis. In hepatocytes, CD36 activation by hexarelin reduces *de novo* cholesterol synthesis. Activation of the LKB-AMPK pathway resulted in the inhibition of the rate-limiting 3-hydroxy-3-methylglutaryl coenzyme A reductase (HMGR) enzyme. Also, induction of Insig1/2 expression through PPARγ/PGC-1α activation led to HMGR degradation and SREBP-2 retention in the endoplasmic reticulum (ER), thereby blunting the homeostatic response to sterol depletion. In adipocytes, CD36 activation with hexarelin promotes mitochondrial activity and biogenesis through enhanced PPARγ and co-activator PGC-1α transcriptional activity. Induction of key genes involved in fatty acid utilization and energy production in mitochondria results in an increased fatty acid β-oxidation and thermogenic-like profile indicative of a browning effect of white fat (adapted from Refs [82,83]).

Besides interacting with GHS-R1a, hexarelin was also identified as a high-affinity ligand for scavenger receptor CD36 based on experiments using rat cardiac membranes [67]. Furthermore, CD36 binding was more specific for hexarelin than other GHRPs, since compounds such as MK-0677 and EP51389 were unable to compete with hexarelin binding. Such findings correlated well with initial observations highlighting the cardioprotective properties of hexarelin in GH-deficient rats [68,69] and the different tissue binding pattern of hexarelin compared to that of MK-0677 or ghrelin [70]. Scavenger receptor CD36 is a surface glycoprotein originally known as fatty acid translocase (FAT). CD36 topology predicts for two transmembrane domains separated by a large extracellular domain with multiple *N*-linked glycosylation sites, and two short cytoplasmic tails required for intracellular signaling (Figure 1). The extracellular loop also contains a proline-rich domain and a hydrophobic stretch thought to loop back into the membrane bilayer. Despite the short length of the two cytoplasmic domains, each contains known sites for modification, such as palmitoylation that guides CD36 to membrane lipid rafts [71] and ubiquitination to sort the receptor for lysosomal degradation [72].

CD36 has been extensively studied for its role in facilitating long-chain fatty acid uptake and oxidation, positioning CD36 as a key player in FA metabolism [73,74,75]. However, its wide expression pattern and numerous identified ligands identify CD36 as a multi-functional receptor. Indeed, CD36 is expressed in a variety of cell types and tissues, including but not limited to adipose tissue, macrophages, platelets, endothelial cells, heart, skeletal muscle and liver. Besides long chain fatty acids, it is recognized by thrombospondin, collagen, malaria-infected erythrocytes, lipolysacharrides, anionic phospholipids, and oxidized lipoproteins (e.g., oxLDL) [76,77,78,79]. Therefore, CD36 can function in a wide range of processes not always related to FA uptake, including apoptosis, angiogenesis, phagocytosis, thrombosis, inflammation, and atherosclerosis [80,81]. Whether all these numerous and seemingly unrelated effects share a common underlying mechanism and/or signaling event associated with CD36 remains unclear. However, adding the GHRPs to the list of high affinity ligands for scavenger receptor CD36 certainly provides an additional layer in CD36 complexity of regulation and most importantly give us new opportunities on the clinical value of GHRPs. 

### CD36 and Atherosclerosis

The original observation that cultured macrophages were able to internalize modified low-density lipoproteins at a much higher rate than native LDL particles, resulting in foam cell transformation, has led to the identification of scavenger receptors [84]. Since then, several scavenger receptors have been identified and classified based on their structural features, their capacity to bind modified LDL particles (e.g., acetylated, oxidized) and their contribution to atherogenesis. Each family member possesses distinct properties, although their ligand-recognition specificity often overlaps, which complicates our understanding of their specific role and downstream actions. However, important physiological roles of scavenger receptors have been identified in body protection from infection, clearance of apoptotic cells and removal of modified lipoproteins that might be potentially harmful. 

Scavenger receptor CD36 is a member of the B subtype that also includes SR-B1, which functions as a receptor that binds high-density lipoproteins (HDL) particles and is involved in the reverse cholesterol pathway. On the other hand, through its strong ability to capture oxLDL, CD36 has clearly been established as a critical component for macrophage foam cell formation and a major pro-atherogenic factor. Atherosclerosis is a complex disease consisting of the infiltration and accumulation of LDL and cellular debris within the intima of medium and large arteries following vascular injury or inflammation [85]. Oxidation of LDL particles (oxLDL) is considered a priming step for the development of the atherosclerotic plaque, with subsequent and excessive engulfment of oxLDL by macrophages, which then become foam cells loaded with lipids resulting in fatty streaks and plaque formation. Activation of macrophages and constant recruitment of immune cells to the inflammatory site results in increased cytokine secretion and continuous oxidation of LDL. OxLDL are no longer recognized by the LDL receptor and become high-affinity ligands for scavenger receptors, principally CD36 present on macrophages [86]. Normally, this process allows macrophages to clear the neointima from the harmful abundance of oxLDL. However, in conditions where macrophages become overwhelmed by oxLDL, unbalanced uptake vs clearance of lipids is taking place, resulting in lipid-laden macrophages or foam cells. Enhanced inflammation, cellular necrosis, and thinning of the fibrotic plaque eventually ensue, leading to plaque rupture and thrombosis.

At the molecular level, internalized oxLDL provide oxidized fatty acids that serve as ligands to PPARγ thereby inducing genes such as CD36 and LXRα (NR1H3) with a subsequent increase in HDL production and reverse cholesterol transport [87]. Therefore, the role of CD36 is central to the pro-atherogenic effect of modified LDL particles. 

Studies using apolipoprotein (apo) E-null mice as a model of fatty streak lesions and atherosclerosis have shown that CD36 was essential in that process. When crossed with apoE-negative mice, CD36 null mice were resistant to developing atherosclerosis [88]. CD36-null murine peritoneal macrophages also exhibited impaired binding and uptake of oxLDL, suggesting that CD36 represents the predominant macrophage receptor for oxLDL [89]. Prior studies in humans had already assessed the critical role of CD36 in the uptake of oxLDL and its abundant and specific expression in atherosclerotic plaques [90,91]. CD36 genetic variants were also identified in humans characterized by high serum triglycerides, low HDL levels, and hyperglycemia with insulin resistance, all considered clinical features of metabolic syndrome [75,92,93]. Patients also demonstrated signs of cardiomyopathy, probably due to impaired uptake of long-chain fatty acids essential to maintaining normal heart function. Population studies have also identified several *CD36* polymorphisms linked to increased risk of metabolic syndrome, acute myocardial infarction and type 2 diabetes [81,94,95,96,97,98], which might support their determination in the context of personalized therapeutic strategies. In particular, polymorphisms found to impair LDL-binding domain of CD36 were correlated with increased cardiovascular risk factors and unstable plaque formation. The potential of CD36 as a therapeutic target for atherosclerosis and other complications of metabolic syndrome is therefore emphasized by our increasing knowledge of its mode of action and certainly warrants the development of novel alternatives aimed to correct for these metabolic defects.

## 6. The GHRP-PPARγ Pathway in Macrophages

Scavenging oxLDL has been defined as a beneficial role of CD36 to liberate intima from cholesterol depots but is also instrumental in early steps of atherogenesis [88,99]. Using conditions to prevent GH release, we have determined that long-term treatment with GHRPs markedly decreased plaque formation in apoE-null mice fed a high-fat diet, a model known to develop atherosclerosis [100,101]. In particular, GHRP EP80317, a CD36 specific ligand, and hexarelin were both potent in strongly reducing atherosclerotic lesion areas [100,101]. The interaction of the GHRPs with CD36 was suggested to initiate an intracellular signaling resulting in the activation of the PPARγ-LXRα-ABC metabolic cascade involved in reverse cholesterol pathway (Figure 1). Treatment of mouse peritoneal macrophages as well as differentiated human THP-1 macrophages with hexarelin resulted in an increase in cholesterol efflux. Such cholesterol removal from cells correlated with a rise in the expression of LXRα, ApoE, ABCA1 and ABCG1, all critical players promoting the HDL-mediated cholesterol efflux pathway. 

Considering that expression of LXRα gene can be upregulated by PPARγ ligands [102] and that oxLDL internalization through CD36 results in PPARγ activation with the entry of oxidized fatty acids [12,103], we thus analyzed the effect of hexarelin on PPARγ transcriptional potential. Using cell-based assays, the interaction of hexarelin with either CD36 or GHS-R1a was shown to induce PPARγ transcriptional potential [101]. In addition, the response to hexarelin was strongly impaired in peritoneal macrophages from PPARγ heterozygote mice, suggesting a critical role of PPARγ. These findings highlight the potential of hexarelin to promote a metabolic cascade involving PPARγ and LXRα as an attempt to efficiently remove oxLDL deleterious actions from the vessel wall and shunt free cholesterol into the HDL reverse cholesterol pathway, thus providing a protective effect in condition of plaque formation in vivo.

The beneficial effect of hexarelin on PPARγ activation appears to be balanced with the coordinated induction of LXRα and downstream target genes achieving optimal lipid efflux. Consistent with this, the activation of PPARγ by hexarelin did not result in an increase in CD36 expression, as opposed to oxLDL-induced PPARγ activity which upregulates CD36, leading to subsequent positive autoregulatory loops being considered pro-atherogenic [87,103]. The exact mechanism for such distinct regulation remains unclear, but we found that the ligand binding domain was not necessary for PPARγ activation by hexarelin, thereby avoiding any effect of exogenous PPARγ ligands (e.g., oxidized fatty acids) that might arise from oxLDL entry. This also supports a role for the N-terminal AF-1 domain that might mediate PPARγ transcriptional activation in response to hexarelin-elicited intracellular transduction pathways. In support of this, PPARγ phosphorylation was strongly induced by hexarelin, providing a molecular basis of PPARγ response to hexarelin signaling [83,101]. GHS-R1a activation by hexarelin also increased PPARγ activity and may therefore suggest a concerted role of GHS-R1a to signal PPARγ [101]. Interestingly, activation of GHS-R1a receptor by hexarelin or its natural ligand ghrelin led to enhanced PPARγ phosphorylation through the coordinated action of Fyn and Akt kinases in macrophages [104]. Whether such concerted response of both GHSR-1a and CD36 receptors is required in the overall beneficial effects of hexarelin on atherosclerosis remains to be further explored.

A more recent study has also described the suppressive effect of hexarelin on plaque formation. Using a vitamin-D3 induced rat model of atherosclerosis, hexarelin was shown to reduce foam cell formation, aortic calcium sedimentation, and vascular smooth muscle cell growth [105]. With its ability to promote ligand-independent PPARγ activation, to interfere with the pro-atherogenic regulatory loop resulting from CD36 upregulation, and to increase overall cholesterol efflux from cells, hexarelin represents a potent regulator to correct for pathological imbalance between sterol uptake and efflux that usually leads to foam cell formation.

## 7. The CD36-PPARγ Axis in Adipocytes

Primary defects in energy balance that produce visceral adiposity are sufficient to result in the development of insulin resistance and vascular disease. Current knowledge has implied a role for fat-derived adipokines, such as leptin, tumor necrosis factor TNFα, adiponectin, adipsin and resistin, as important regulators of insulin sensitivity, defining fat tissue not just as a passive storage depot but also as an endocrine organ [106,107]. PPARγ is recognized as a major regulator of adipokine synthesis in mature adipocytes and as such, it has become a therapeutic target of TZD actions [19,20]. Because of their potent insulin-sensitizing activity, TZDs are currently used to correct circulating glucose levels in type 2 diabetes patients but under increasing restricted conditions [9,21,22,23]. 

Activation of PPARγ in white adipocytes is known to promote FA storage, triglyceride (TG) synthesis and glucose uptake involving upregulation of key target genes related to fatty acid metabolism. In addition, the induction of expression and secretion of insulin-sensitizing adipokines, such as adiponectin, will dictate a decrease in lipid accumulation and an increase of glucose uptake and fatty acid oxidation in other tissues. These actions are part of the mechanism by which the TZDs improve insulin resistance in diabetic patients [23]. PPARγ is also a master regulator of adipogenesis. Studies of fat-specific PPARγ knockout mice revealed that PPARγ is essential for differentiation and survival of fat cells [108,109]. Consistent with the dual effect of PPARγ to ameliorate insulin sensitivity while promoting fat differentiation, genetic studies have revealed that a partial loss-of-function Pro12Ala variant improved insulin sensitivity, while the gain-of-function Pro115Gln mutation was associated to obesity and insulin resistance in humans [110]. Therefore, it becomes essential to consider a PPARγ selective modulator that might exhibit a better insulin sensitizing profile as compared to a full agonist.

Several studies have shown that mature adipocytes do express CD36, whereas expression of GHS-R1a remains unclear despite a functional response to ghrelin [111,112]. However, the mechanism by which CD36 may affect the overall metabolic activity of fat storage and mobilization is not completely defined. Based on evidence that CD36 activation with hexarelin resulted in PPARγ activation in macrophages, it was expected that a similar activation of PPARγ and subsequent downstream effects could take place in adipocytes. 

Indeed, we found that hexarelin promoted beneficial effects in white adipose tissue, resulting in a striking thermogenic profile of FA oxidation and mitochondria biogenesis in cultured adipocytes and in epididymal fat of treated mice [113]. These effects were translated through PPARγ and required CD36, establishing a functional CD36-PPARγ pathway in fat [83]. Interestingly, gene profile analysis has revealed that many of the genes upregulated by hexarelin were shared with TZD treatment, indicating a common effect on PPARγ activation. However, not all established PPARγ targets were upregulated by hexarelin, including CD36 itself [113]. This was also observed in macrophages, suggesting a similar mechanism for CD36 gene regulation in response to hexarelin (Figure 1). Gene expression and functional studies have indicated that adipocytes respond to hexarelin with an increased mobilization of fatty acids rather than the expected adipogenic effect of PPARγ activation seen with TZDs, revealing an unexpected effect of hexarelin to promote the β-oxidation of fatty acids [113]. Whether this indicates that hexarelin may serve as an energy deficit signal that prevents fat utilization during deprivation and promotes its use in excess is not certain, but if true, such a scenario has clear implications for obesity-related metabolic defects. Consistent with this, the induction of key markers of fatty acid oxidation and mitochondrial activity, including Cpt1b, Acaa1 and 2, and several subunits of the cytochrome c oxidase (COX) complex, were increased in response to hexarelin. Interestingly, a recent study has implicated the metabolic response of white fat tissue to hexarelin in correcting abnormal lipid metabolic states of insulin-resistant mice through modulation of genes related to fatty acid uptake and oxidation [114]. Given that PPARα also plays a pivotal role in FA metabolism by regulating genes related to mitochondrial and peroxisomal β-oxidation pathways in high oxidative tissues, such as liver, heart and brown fat [115,116], the metabolic response of fat to hexarelin strongly suggests also a role for PPARα activation. Consistent with this, we found that both PPARα and PPARβ/δ were activated in response to hexarelin, supporting a cellular response to CD36 activation that might implicate the various PPAR isotypes [83,101]. 

The preferred redirection of FA toward mitochondrial oxidation process was accompanied by noticeable changes in mitochondrial morphology in white adipose tissue of hexarelin-treated mice. Increases in the intramitochondrial matrix surface and cristae formation observed were typical of tissues with high oxidative potential, such as brown fat, suggesting a browning effect of hexarelin [113]. This was also consistent with the induction of key thermogenic markers PGC-1α and uncoupling protein (UCP)-1, which rose from low normal levels usually found in white fat cells to those mainly characteristic of brown fat. PGC-1α and UCP-1 are highly expressed in brown fat and play critical roles in thermogenesis and energy expenditure with enhanced oxidative metabolism and mitochondrial biogenesis [117,118,119]. The ability of hexarelin to upregulate PGC-1α provides a clue by which CD36 signaling might control the fine-tuning of mitochondrial function towards FA oxidation and energy balance. This suggests that such increase in mitochondrial activity and biogenesis by hexarelin might thus provide a benefit to defects associated to mitochondrial diseases. Consistent with our findings, a recent study also reported a protective effect of hexarelin on mitochondria function using a rat model of cachexia [120]. The authors reported an increase of mitochondrial markers such as PGC-1α at the protein levels, supporting the potential of hexarelin to induce a mitochondrial response, but the mechanism involved and the role of CD36 were not addressed in this context. Interestingly, besides PPARγ, PGC-1α upregulation by hexarelin and CD36 activation might also affect other known nuclear receptors coregulated by PGC-1, such as the estrogen-related receptors (ERRs) involved in mitochondrial function and biogenesis [121,122,123]. Therefore, investigating their contribution is certainly an interesting avenue to pursue.

## 8. The Hexarelin-PPARγ Axis in Hepatocytes

Although considered highly expressed in insulin-sensitizing tissues, PPARγ is found at low levels in the liver, and therefore its influence on hepatic function is not fully understood. In fact, much negative attention was given to hepatic PPARγ with the hepatotoxicity effect of TZD troglitazone, resulting in its withdrawal from the market [26,27]. Part of the noxious effects of troglitazone in liver was associated with the production of toxic reactive metabolites and signs of mitochondrial DNA damage, mitochondrial defects and cell death [124,125], which emphasizes anti-oxidant strategies [126]. However, some evidence indicates that the toxic effect of troglitazone might be independent of PPARγ activity [127]. Recent studies have reported beneficial hepatic effects of PPARγ agonists in reversing nonalcoholic steatohepatitis (NASH) in patients, reducing liver inflammation, fibrosis and triglyceride content [128,129]. Interestingly, in condition of PPARγ overexpression triggered by insulin or oleic acid treatment in hepatocytes, or induced in mice fed a high-fat diet, there was the expected increase in PPARγ lipogenic genes but also of PPARα target genes involved in FA oxidation [130,131,132]. Such induction of hepatic PPARγ might therefore represent an adaptive response to promote beneficial lipid utilization. 

The role of GHRPs on liver function has not been fully characterized and given their ability to promote macrophage cholesterol reverse transport through CD36 receptor, one would expect that the CD36-PPARγ axis might play a role of regulation on sterol metabolism in hepatic cells. We have recently reported that hexarelin regulates hepatic cholesterol homeostasis by repressing de novo cholesterol synthesis through enhanced 3-hydroxy-3-methylglutaryl coenzyme A reductase (HMGR) degradation and sterol regulatory element-binding protein (SREBP)-2 retention in the endoplasmic reticulum [82]. Elegant work from Brown and Goldstein has detailed the mechanism responsible for maintaining hepatic cholesterol homeostasis [133,134]. The rate-limiting HMGR is under a tight control by available cellular cholesterol content both at the gene level, through regulation of expression by sterol regulatory element-binding protein SREBP-2, and at the protein level, through enzyme phosphorylation and degradation. Our findings have demonstrated that CD36 activity reduced cholesterol levels in liver cells by impeding the compensatory activation of HMGR and decreasing SREBP-2 transactivation normally occurring in cells during sterol depletion (Figure 1). Interestingly, this potential of CD36 to inhibit cholesterol synthesis was associated with activation of the LKB/AMPK energetic pathway, known to play an imperative role in energy homeostasis by regulating a plethora of pathways for the main purpose of saving energy and access readily available fuel for the cell. The AMPK activation by hexarelin resulted in the phosphorylation of HMGR, achieving a rapid inhibition of its activity in hepatocytes, similar to the inhibition triggered by statin compounds. With the role of CD36 in internalizing long chain fatty acids and cholesterol derivatives, the immediate activation of AMPK by hexarelin is believed to promote a need to preserve energy in liver cells. Similarly, fatty acid-induced AMPK activation has also been reported in the heart to promote CD36 regulation and adjust for fatty acid usage and oxidation [135,136]. Although the exact role remains to be determined, our findings suggest a metabolic cascade between CD36 and the LKB/AMPK pathway, providing a role of CD36 to regulate downstream AMPK targets involved in energy metabolism.

The CD36-PPARγ pathway appears to be functional in hepatocytes with the activation of PPARγ by hexarelin, which identified Insig-1 and Insig-2 genes as PPARγ-responsive genes [82]. Insig-1 and Insig-2 were reported to promote HMGR ubiquitination and degradation [137], and also to prevent the transit of SREBP-2 to the Golgi for its processing and activation [133,138]. Therefore, this provides a mechanism by which genes encoding key enzymes involved in cholesterol synthesis and under the control of SREBP-2 remained unresponsive to sterol depletion in the context of CD36 activation by hexarelin [82]. The rapid Insig-mediated degradation of HMGR protein and the retention of SREBP-2 in the endoplasmic reticulum represent two checkpoints of regulation of CD36 signaling to prevent sterol accumulation in liver cells.

Interestingly, the coactivation potential of PGC-1α was enhanced in response to hexarelin, accompanied by an increase in PGC-1α recruitment to PPARγ [82]. This suggests that CD36 can signal PGC-1α to induce PPARγ coactivation in hepatocytes. Consistent with this, the recruitment of PGC-1α to activated AMPKα was enhanced by hexarelin, leading to Sirt1-mediated deacetylation and PGC-1α transcriptional activation. Such metabolic activation of PGC-1α has also been described in adipocytes whereby CD36 promoted increases in PGC-1α and downstream effectors, such as UCP-1 and ATP synthase [113]. Given a similar increase in PGC-1α activity and UCP-1 expression in hepatocytes, and the prominent role of PGC-1 in cellular energy homeostasis, FA oxidation, hepatic gluconeogenesis, and mitochondrial biogenesis [117,139,140], the role of CD36 is likely to be extended to different pathways of regulation involved in liver metabolism and function.

## 9. Concluding Remarks

While the molecular events by which CD36 and GHRPs exert their actions are not completely understood, increasing evidence supports a prominent role of scavenger receptor CD36 to initiate profound changes in key metabolic pathways, especially pertaining to PPARγ-controlled critical steps. Also, with its potential to promote PGC-1α transcriptional competence and related key functions of fatty acid usage, glucose homeostasis and mitochondrial activity, we might expect that GHRP-CD36 signaling may expand to other metabolic pathways and involve additional nuclear receptors. Given the increasing prevalence of metabolic defects associated with deregulated glucose and lipid metabolism and with mitochondria dysfunction, targeting CD36 with GHRPs appears to be a safe option for the treatment of metabolic disorders.
